# Supplementary material for: HIVprotI: an integrated web based platform for prediction and design of HIV proteins inhibitors
Source: J Cheminform. 2018 Mar 9;10:12. doi: 10.1186/s13321-018-0266-y (PMC5845081; doi:10.1186/s13321-018-0266-y)
Supplement: Supplementary file 2 — Additional file 2. Source code of HIVProtI web server. [file 13321_2018_266_MOESM2_ESM.zip › HIVprotI_Source-code/hivprotI/weka-3-6-6/doc/weka/experiment/PairedTTester.html]

PairedTTester


---


|  |  |  |
| --- | --- | --- |
| **Overview** \| **Package** \| **Class** \| **Tree** \| **Deprecated** \| **Index** \| **Help** \| **Weka's home** | |  |
| **PREV CLASS**   **NEXT CLASS** | **FRAMES**    **NO FRAMES**     **All Classes** |  |
| SUMMARY: NESTED \| FIELD \| CONSTR \| METHOD | DETAIL: FIELD \| CONSTR \| METHOD |  |


---


## weka.experiment Class PairedTTester

```
java.lang.Object
  weka.experiment.PairedTTester
```

**All Implemented Interfaces:**: java.io.Serializable, OptionHandler, RevisionHandler, Tester

**Direct Known Subclasses:**: PairedCorrectedTTester

---

``` public class PairedTTester extends java.lang.Object implements OptionHandler, Tester, RevisionHandler ```

Calculates T-Test statistics on data stored in a set of instances.


Valid options are:

```
 -D <index,index2-index4,...>
  Specify list of columns that specify a unique
  dataset.
  First and last are valid indexes. (default none)
```

```
 -R <index>
  Set the index of the column containing the run number
```

```
 -F <index>
  Set the index of the column containing the fold number
```

```
 -G <index1,index2-index4,...>
  Specify list of columns that specify a unique
  'result generator' (eg: classifier name and options).
  First and last are valid indexes. (default none)
```

```
 -S <significance level>
  Set the significance level for comparisons (default 0.05)
```

```
 -V
  Show standard deviations
```

```
 -L
  Produce table comparisons in Latex table format
```

```
 -csv
  Produce table comparisons in CSV table format
```

```
 -html
  Produce table comparisons in HTML table format
```

```
 -significance
  Produce table comparisons with only the significance values
```

```
 -gnuplot
  Produce table comparisons output suitable for GNUPlot
```

**Version:**
:   $Revision: 6431 $

**Author:**
:   Len Trigg (trigg@cs.waikato.ac.nz)

**See Also:**: Serialized Form

---

**Constructor Summary**

`PairedTTester()`


| **Method Summary** | |
| --- | --- |
| `void` | `assign(Tester tester)`             retrieves all the settings from the given Tester |
| `PairedStats` | `calculateStatistics(Instance datasetSpecifier, int resultset1Index, int resultset2Index, int comparisonColumn)`             Computes a paired t-test comparison for a specified dataset between two resultsets. |
| `boolean` | `displayResultset(int index)`             Checks whether the resultset with the given index shall be displayed. |
| `Range` | `getDatasetKeyColumns()`             Get the value of DatasetKeyColumns. |
| `int[]` | `getDisplayedResultsets()`             Gets the indices of the the datasets that are displayed (if `null` then all are displayed). |
| `java.lang.String` | `getDisplayName()`             returns the name of the tester |
| `int` | `getFoldColumn()`             Get the value of FoldColumn. |
| `Instances` | `getInstances()`             Get the value of Instances. |
| `int` | `getNumDatasets()`             Gets the number of datasets in the resultsets |
| `int` | `getNumResultsets()`             Gets the number of resultsets in the data. |
| `java.lang.String[]` | `getOptions()`             Gets current settings of the PairedTTester. |
| `ResultMatrix` | `getResultMatrix()`             Gets the instance that produces the output. |
| `Range` | `getResultsetKeyColumns()`             Get the value of ResultsetKeyColumns. |
| `java.lang.String` | `getResultsetName(int index)`             Gets a string descriptive of the specified resultset. |
| `java.lang.String` | `getRevision()`             Returns the revision string. |
| `int` | `getRunColumn()`             Get the value of RunColumn. |
| `boolean` | `getShowStdDevs()`             Returns true if standard deviations have been requested. |
| `double` | `getSignificanceLevel()`             Get the value of SignificanceLevel. |
| `int` | `getSortColumn()`             Returns the column to sort on, -1 means the default sorting. |
| `java.lang.String` | `getSortColumnName()`             Returns the name of the column to sort on. |
| `java.lang.String` | `getToolTipText()`             returns a string that is displayed as tooltip on the "perform test" button in the experimenter |
| `java.lang.String` | `header(int comparisonColumn)`             Creates a "header" string describing the current resultsets. |
| `java.util.Enumeration` | `listOptions()`             Lists options understood by this object. |
| `static void` | `main(java.lang.String[] args)`             Test the class from the command line. |
| `java.lang.String` | `multiResultsetFull(int baseResultset, int comparisonColumn)`             Creates a comparison table where a base resultset is compared to the other resultsets. |
| `java.lang.String` | `multiResultsetRanking(int comparisonColumn)`             returns a ranking of the resultsets |
| `java.lang.String` | `multiResultsetSummary(int comparisonColumn)`             Carries out a comparison between all resultsets, counting the number of datsets where one resultset outperforms the other. |
| `int[][]` | `multiResultsetWins(int comparisonColumn, int[][] nonSigWin)`             Carries out a comparison between all resultsets, counting the number of datsets where one resultset outperforms the other. |
| `java.lang.String` | `resultsetKey()`             Creates a key that maps resultset numbers to their descriptions. |
| `void` | `setDatasetKeyColumns(Range newDatasetKeyColumns)`             Set the value of DatasetKeyColumns. |
| `void` | `setDisplayedResultsets(int[] cols)`             Sets the indicies of the datasets to display (`null` means all). |
| `void` | `setFoldColumn(int newFoldColumn)`             Set the value of FoldColumn. |
| `void` | `setInstances(Instances newInstances)`             Set the value of Instances. |
| `void` | `setOptions(java.lang.String[] options)`             Parses a given list of options. |
| `void` | `setResultMatrix(ResultMatrix matrix)`             Sets the matrix to use to produce the output. |
| `void` | `setResultsetKeyColumns(Range newResultsetKeyColumns)`             Set the value of ResultsetKeyColumns. |
| `void` | `setRunColumn(int newRunColumn)`             Set the value of RunColumn. |
| `void` | `setShowStdDevs(boolean s)`             Set whether standard deviations are displayed or not. |
| `void` | `setSignificanceLevel(double newSignificanceLevel)`             Set the value of SignificanceLevel. |
| `void` | `setSortColumn(int newSortColumn)`             Set the column to sort on, -1 means the default sorting. |

**Methods inherited from class java.lang.Object**

`equals, getClass, hashCode, notify, notifyAll, toString, wait, wait, wait`

**Constructor Detail**

### PairedTTester

```
public PairedTTester()
```


**Method Detail**

### setResultMatrix

```
public void setResultMatrix(ResultMatrix matrix)
```

:   Sets the matrix to use to produce the output.

    :   **Specified by:**: `setResultMatrix` in interface `Tester`
    :   **Parameters:**: `matrix` - the instance to use to produce the output **See Also:**: `ResultMatrix`

---


### getResultMatrix

```
public ResultMatrix getResultMatrix()
```

:   Gets the instance that produces the output.

    :   **Specified by:**: `getResultMatrix` in interface `Tester`
    :   **Returns:**: the instance to produce the output

---


### setShowStdDevs

```
public void setShowStdDevs(boolean s)
```

:   Set whether standard deviations are displayed or not.

    :   **Specified by:**: `setShowStdDevs` in interface `Tester`
    :   **Parameters:**: `s` - true if standard deviations are to be displayed

---


### getShowStdDevs

```
public boolean getShowStdDevs()
```

:   Returns true if standard deviations have been requested.

    :   **Specified by:**: `getShowStdDevs` in interface `Tester`
    :   **Returns:**: true if standard deviations are to be displayed.

---


### getNumDatasets

```
public int getNumDatasets()
```

:   Gets the number of datasets in the resultsets

    :   **Specified by:**: `getNumDatasets` in interface `Tester`
    :   **Returns:**: the number of datasets in the resultsets

---


### getNumResultsets

```
public int getNumResultsets()
```

:   Gets the number of resultsets in the data.

    :   **Specified by:**: `getNumResultsets` in interface `Tester`
    :   **Returns:**: the number of resultsets in the data

---


### getResultsetName

```
public java.lang.String getResultsetName(int index)
```

:   Gets a string descriptive of the specified resultset.

    :   **Specified by:**: `getResultsetName` in interface `Tester`
    :   **Parameters:**: `index` - the index of the resultset **Returns:**: a descriptive string for the resultset

---


### displayResultset

```
public boolean displayResultset(int index)
```

:   Checks whether the resultset with the given index shall be displayed.

    :   **Specified by:**: `displayResultset` in interface `Tester`
    :   **Parameters:**: `index` - the index of the resultset to check whether it shall be displayed **Returns:**: whether the specified resultset is displayed

---


### calculateStatistics

```
public PairedStats calculateStatistics(Instance datasetSpecifier,
                                       int resultset1Index,
                                       int resultset2Index,
                                       int comparisonColumn)
                                throws java.lang.Exception
```

:   Computes a paired t-test comparison for a specified dataset between
    two resultsets.

    :   **Specified by:**: `calculateStatistics` in interface `Tester`
    :   **Parameters:**: `datasetSpecifier` - the dataset specifier: `resultset1Index` - the index of the first resultset: `resultset2Index` - the index of the second resultset: `comparisonColumn` - the column containing values to compare **Returns:**: the results of the paired comparison **Throws:**: `java.lang.Exception` - if an error occurs

---


### resultsetKey

```
public java.lang.String resultsetKey()
```

:   Creates a key that maps resultset numbers to their descriptions.

    :   **Specified by:**: `resultsetKey` in interface `Tester`
    :   **Returns:**: a value of type 'String'

---


### header

```
public java.lang.String header(int comparisonColumn)
```

:   Creates a "header" string describing the current resultsets.

    :   **Specified by:**: `header` in interface `Tester`
    :   **Parameters:**: `comparisonColumn` - a value of type 'int' **Returns:**: a value of type 'String'

---


### multiResultsetWins

```
public int[][] multiResultsetWins(int comparisonColumn,
                                  int[][] nonSigWin)
                           throws java.lang.Exception
```

:   Carries out a comparison between all resultsets, counting the number
    of datsets where one resultset outperforms the other.

    :   **Specified by:**: `multiResultsetWins` in interface `Tester`
    :   **Parameters:**: `comparisonColumn` - the index of the comparison column: `nonSigWin` - for storing the non-significant wins **Returns:**: a 2d array where element [i][j] is the number of times resultset j performed significantly better than resultset i. **Throws:**: `java.lang.Exception` - if an error occurs

---


### multiResultsetSummary

```
public java.lang.String multiResultsetSummary(int comparisonColumn)
                                       throws java.lang.Exception
```

:   Carries out a comparison between all resultsets, counting the number
    of datsets where one resultset outperforms the other. The results
    are summarized in a table.

    :   **Specified by:**: `multiResultsetSummary` in interface `Tester`
    :   **Parameters:**: `comparisonColumn` - the index of the comparison column **Returns:**: the results in a string **Throws:**: `java.lang.Exception` - if an error occurs

---


### multiResultsetRanking

```
public java.lang.String multiResultsetRanking(int comparisonColumn)
                                       throws java.lang.Exception
```

:   returns a ranking of the resultsets

    :   **Specified by:**: `multiResultsetRanking` in interface `Tester`
    :   **Parameters:**: `comparisonColumn` - the column to compare with **Returns:**: the ranking **Throws:**: `java.lang.Exception` - if something goes wrong

---


### multiResultsetFull

```
public java.lang.String multiResultsetFull(int baseResultset,
                                           int comparisonColumn)
                                    throws java.lang.Exception
```

:   Creates a comparison table where a base resultset is compared to the
    other resultsets. Results are presented for every dataset.

    :   **Specified by:**: `multiResultsetFull` in interface `Tester`
    :   **Parameters:**: `baseResultset` - the index of the base resultset: `comparisonColumn` - the index of the column to compare over **Returns:**: the comparison table string **Throws:**: `java.lang.Exception` - if an error occurs

---


### listOptions

```
public java.util.Enumeration listOptions()
```

:   Lists options understood by this object.

    :   **Specified by:**: `listOptions` in interface `OptionHandler`
    :   **Returns:**: an enumeration of Options.

---


### setOptions

```
public void setOptions(java.lang.String[] options)
                throws java.lang.Exception
```

:   Parses a given list of options.


    Valid options are:

    ```
     -D <index,index2-index4,...>
      Specify list of columns that specify a unique
      dataset.
      First and last are valid indexes. (default none)
    ```

    ```
     -R <index>
      Set the index of the column containing the run number
    ```

    ```
     -F <index>
      Set the index of the column containing the fold number
    ```

    ```
     -G <index1,index2-index4,...>
      Specify list of columns that specify a unique
      'result generator' (eg: classifier name and options).
      First and last are valid indexes. (default none)
    ```

    ```
     -S <significance level>
      Set the significance level for comparisons (default 0.05)
    ```

    ```
     -V
      Show standard deviations
    ```

    ```
     -L
      Produce table comparisons in Latex table format
    ```

    ```
     -csv
      Produce table comparisons in CSV table format
    ```

    ```
     -html
      Produce table comparisons in HTML table format
    ```

    ```
     -significance
      Produce table comparisons with only the significance values
    ```

    ```
     -gnuplot
      Produce table comparisons output suitable for GNUPlot
    ```

    :   **Specified by:**: `setOptions` in interface `OptionHandler`
    :   **Parameters:**: `options` - an array containing options to set. **Throws:**: `java.lang.Exception` - if invalid options are given

---


### getOptions

```
public java.lang.String[] getOptions()
```

:   Gets current settings of the PairedTTester.

    :   **Specified by:**: `getOptions` in interface `OptionHandler`
    :   **Returns:**: an array of strings containing current options.

---


### getResultsetKeyColumns

```
public Range getResultsetKeyColumns()
```

:   Get the value of ResultsetKeyColumns.

    :   **Specified by:**: `getResultsetKeyColumns` in interface `Tester`
    :   **Returns:**: Value of ResultsetKeyColumns.

---


### setResultsetKeyColumns

```
public void setResultsetKeyColumns(Range newResultsetKeyColumns)
```

:   Set the value of ResultsetKeyColumns.

    :   **Specified by:**: `setResultsetKeyColumns` in interface `Tester`
    :   **Parameters:**: `newResultsetKeyColumns` - Value to assign to ResultsetKeyColumns.

---


### getDisplayedResultsets

```
public int[] getDisplayedResultsets()
```

:   Gets the indices of the the datasets that are displayed (if `null`
    then all are displayed). The base is always displayed.

    :   **Specified by:**: `getDisplayedResultsets` in interface `Tester`
    :   **Returns:**: the indices of the datasets to display

---


### setDisplayedResultsets

```
public void setDisplayedResultsets(int[] cols)
```

:   Sets the indicies of the datasets to display (`null` means all).
    The base is always displayed.

    :   **Specified by:**: `setDisplayedResultsets` in interface `Tester`
    :   **Parameters:**: `cols` - the indices of the datasets to display

---


### getSignificanceLevel

```
public double getSignificanceLevel()
```

:   Get the value of SignificanceLevel.

    :   **Specified by:**: `getSignificanceLevel` in interface `Tester`
    :   **Returns:**: Value of SignificanceLevel.

---


### setSignificanceLevel

```
public void setSignificanceLevel(double newSignificanceLevel)
```

:   Set the value of SignificanceLevel.

    :   **Specified by:**: `setSignificanceLevel` in interface `Tester`
    :   **Parameters:**: `newSignificanceLevel` - Value to assign to SignificanceLevel.

---


### getDatasetKeyColumns

```
public Range getDatasetKeyColumns()
```

:   Get the value of DatasetKeyColumns.

    :   **Specified by:**: `getDatasetKeyColumns` in interface `Tester`
    :   **Returns:**: Value of DatasetKeyColumns.

---


### setDatasetKeyColumns

```
public void setDatasetKeyColumns(Range newDatasetKeyColumns)
```

:   Set the value of DatasetKeyColumns.

    :   **Specified by:**: `setDatasetKeyColumns` in interface `Tester`
    :   **Parameters:**: `newDatasetKeyColumns` - Value to assign to DatasetKeyColumns.

---


### getRunColumn

```
public int getRunColumn()
```

:   Get the value of RunColumn.

    :   **Specified by:**: `getRunColumn` in interface `Tester`
    :   **Returns:**: Value of RunColumn.

---


### setRunColumn

```
public void setRunColumn(int newRunColumn)
```

:   Set the value of RunColumn.

    :   **Specified by:**: `setRunColumn` in interface `Tester`
    :   **Parameters:**: `newRunColumn` - Value to assign to RunColumn.

---


### getFoldColumn

```
public int getFoldColumn()
```

:   Get the value of FoldColumn.

    :   **Specified by:**: `getFoldColumn` in interface `Tester`
    :   **Returns:**: Value of FoldColumn.

---


### setFoldColumn

```
public void setFoldColumn(int newFoldColumn)
```

:   Set the value of FoldColumn.

    :   **Specified by:**: `setFoldColumn` in interface `Tester`
    :   **Parameters:**: `newFoldColumn` - Value to assign to FoldColumn.

---


### getSortColumnName

```
public java.lang.String getSortColumnName()
```

:   Returns the name of the column to sort on.

    :   **Specified by:**: `getSortColumnName` in interface `Tester`
    :   **Returns:**: the name of the column to sort on.

---


### getSortColumn

```
public int getSortColumn()
```

:   Returns the column to sort on, -1 means the default sorting.

    :   **Specified by:**: `getSortColumn` in interface `Tester`
    :   **Returns:**: the column to sort on.

---


### setSortColumn

```
public void setSortColumn(int newSortColumn)
```

:   Set the column to sort on, -1 means the default sorting.

    :   **Specified by:**: `setSortColumn` in interface `Tester`
    :   **Parameters:**: `newSortColumn` - the new sort column.

---


### getInstances

```
public Instances getInstances()
```

:   Get the value of Instances.

    :   **Specified by:**: `getInstances` in interface `Tester`
    :   **Returns:**: Value of Instances.

---


### setInstances

```
public void setInstances(Instances newInstances)
```

:   Set the value of Instances.

    :   **Specified by:**: `setInstances` in interface `Tester`
    :   **Parameters:**: `newInstances` - Value to assign to Instances.

---


### assign

```
public void assign(Tester tester)
```

:   retrieves all the settings from the given Tester

    :   **Specified by:**: `assign` in interface `Tester`
    :   **Parameters:**: `tester` - the Tester to get the settings from

---


### getToolTipText

```
public java.lang.String getToolTipText()
```

:   returns a string that is displayed as tooltip on the "perform test"
    button in the experimenter

    :   **Specified by:**: `getToolTipText` in interface `Tester`
    :   **Returns:**: the tool tip

---


### getDisplayName

```
public java.lang.String getDisplayName()
```

:   returns the name of the tester

    :   **Specified by:**: `getDisplayName` in interface `Tester`
    :   **Returns:**: the display name

---


### getRevision

```
public java.lang.String getRevision()
```

:   Returns the revision string.

    :   **Specified by:**: `getRevision` in interface `RevisionHandler`
    :   **Returns:**: the revision

---


### main

```
public static void main(java.lang.String[] args)
```

:   Test the class from the command line.

    :   **Parameters:**: `args` - contains options for the instance ttests


---


|  |  |  |
| --- | --- | --- |
| **Overview** \| **Package** \| **Class** \| **Tree** \| **Deprecated** \| **Index** \| **Help** \| **Weka's home** | |  |
| **PREV CLASS**   **NEXT CLASS** | **FRAMES**    **NO FRAMES**     **All Classes** |  |
| SUMMARY: NESTED \| FIELD \| CONSTR \| METHOD | DETAIL: FIELD \| CONSTR \| METHOD |  |


---
